# The Relationship between Prenatal PCB Exposure and Intelligence (IQ) in 9-Year-Old Children

**DOI:** 10.1289/ehp.11058

**Published:** 2008-05-28

**Authors:** Paul W. Stewart, Edward Lonky, Jacqueline Reihman, James Pagano, Brooks B. Gump, Thomas Darvill

**Affiliations:** 1 Center for Neurobehavioral Effects of Environmental Toxics and; 2 Environmental Research Center, State University of New York at Oswego, Oswego, New York, USA

**Keywords:** children, cognitive, intelligence, IQ, mercury, Oswego, PCBs, polychlorinated biphenyls, Wechsler, WISC-III

## Abstract

**Background:**

Several epidemiologic studies have demonstrated relationships between prenatal exposure to polychlorinated biphenyls (PCBs) and modest cognitive impairments in infancy and early childhood. However, few studies have followed cohorts of exposed children long enough to examine the possible impact of prenatal PCB exposure on psychometric intelligence in later childhood. Of the few studies that have done so, one in the Great Lakes region of the United States reported impaired IQ in children prenatally exposed to PCBs, whereas another found no association.

**Objectives:**

This study was designed to determine whether environmental exposure to PCBs predicts lower IQ in school-age children in the Great Lakes region of the northeastern United States.

**Methods:**

We measured prenatal exposure to PCBs and IQ at 9 years of age in 156 subjects from Oswego, New York. We also measured > 50 potential predictors of intelligence in children, including repeated measures of the home environment [Home Observation for Measurement of the Environment (HOME)], socioeconomic status (SES), parental IQ, alcohol/cigarette use, neonatal risk factors, and nutrition.

**Results:**

For each 1-ng/g (wet weight) increase in PCBs in placental tissue, Full Scale IQ dropped by three points (*p* = 0.02), and Verbal IQ dropped by four points (*p* = 0.003). The median PCB level was 1.50 ng/g, with a lower quartile of 1.00 ng/g and an upper quartile of 2.06 ng/g. Moreover, this association was significant after controlling for many potential confounders, including prenatal exposure to methylmercury, dichlorodiphenyldichloroethylene, hexachlorobenzene, and lead.

**Conclusions:**

These results, in combination with similar results obtained from a similar study in the Great Lakes conducted 10 years earlier, indicate that prenatal PCB exposure in the Great Lakes region is associated with lower IQ in children.

Several studies have been conducted to investigate the relationship between prenatal exposure to polychlorinated biphenyls (PCBs) and cognitive development in children (Jacobson and Jacobson 1996, 2003; Stewart et al. 2003a, 2003b; Walkowiak et al. 2001). Most studies have reported deleterious associations between prenatal PCB exposure and behavioral development (Darvill et al. 2000; Jacobson and Jacobson 1997; Jacobson et al. 1985; Patandin et al. 1999; Stewart et al. 2003a, 2003b, 2005b, 2006). As valuable as these studies have been in determining the potential neurobehavioral toxicity of PCBs, very few have investigated long-term associations between PCBs and cognitive behaviors that have documented financial consequences (Grosse et al. 2002; Salkever 1995; Schwartz 1994) across the life span. Psychometric intelligence (IQ) is arguably one of the most widely used end points for functional consequences to both the individual and society. The IQ score is considered a measure of aptitude and a predictor of school achievement, and is useful as a clinical and educational diagnostic tool.

Of all the longitudinal PCB studies to date, only two (Gray et al. 2005; Jacobson and Jacobson 1996) have examined psychometric intelligence in PCB-exposed children. Jacobson and Jacobson (1996) found that prenatal exposure to PCBs was predictive of impaired IQ [Wechsler Intelligence Scale for Children, Revised (WISC-R)] in 11-year-old children living in the Great Lakes region (Michigan) of the United States. This association was particularly strong with respect to Verbal Intelligence and the Freedom from Distractibility subscale. In contrast to the Jacobson and Jacobson (1996) findings, Gray et al. (2005) did not find an association between PCBs and intelligence. A notable difference between the two studies was that the former included a sample of children exposed to PCBs from maternal consumption of PCB-contaminated Lake Michigan sport fish, and the latter examined prenatal PCB exposure in the general population. Differences in the routes of exposure, as well as possible differences in the pattern of PCB congeners in the Great Lakes (Stewart et al. 1999, 2000a), may account for the discrepant results. For instance, although PCB levels in the Oswego cohort in the 1990s are far lower than exposure levels in the Collaborative Perinatal Project (Longnecker et al. 2003), PCB consumption through fish in the Oswego cohort is associated with a more highly chlorinated pattern of PCBs (Stewart et al. 1999) as well as higher acute dose “pulses” resulting from recent fish consumption (Humphrey 1987; Stewart et al. 1999). Nevertheless, conflicting results on PCBs and IQ leave the issue concerning PCB effects on IQ far from resolved, and there can be little debate that more data are needed.

As described elsewhere (Lonky et al. 1996; Stewart et al. 2006), the Oswego cohort was designed to replicate and extend the findings of the Lake Michigan study. Data collected over the past decade have demonstrated a remarkably similar pattern of findings between the Oswego and Michigan cohorts, characterized by cross-cohort replications of PCB-related impairments in early neonatal behavior (Jacobson et al. 1984; Stewart et al. 2000b), infant visual recognition memory (Darvill et al. 2000; Jacobson et al. 1985), early childhood cognitive abilities (Jacobson et al. 1990, Stewart et al. 2003b), and impaired impulse control (Jacobson and Jacobson 2003; Stewart et al. 2003a, 2005a, 2006).

The current study was designed to determine whether the relationship between prenatal PCB exposure and IQ observed in the Lake Michigan cohort would be replicable in a different cohort of PCB-exposed children in the Lake Ontario basin (Lonky et al. 1996). Pursuant to this goal, we examined the relationship between prenatal PCB levels to post-natal IQ at 9 years of age. Based on the findings by Jacobson and Jacobson (1996) in Lake Michigan, we hypothesized that prenatal PCB exposure would predict impaired Full Scale IQ. We also predicted a pattern similar to the Jacobson and Jacobson (1996) findings that PCBs would be significantly related to Verbal IQ and Freedom from Distractibility, but not to Performance IQ.

## Materials and Methods

### Subjects

The Oswego study tracks a cohort of children that were born between 1991 and 1994. For the present report, we tested all children near their birthday at 9 years (±2 months) of age (Table 1).

Mothers and their children that are currently enrolled in the Oswego study participated as part of an ongoing, longitudinal study of the relationship between prenatal PCB exposure and cognitive development in children. This study complied with all U.S. regulations [i.e., institutional review board (IRB) research approval] regarding human subjects, and we obtained IRB-approved written informed consent for all subjects before participation in the study. The sampling methodology and demographic and exposure characteristics of this cohort have been previously published in detail (Lonky et al. 1996; Stewart et al. 1999, 2000b). Of the 293 original enrollees for whom cord blood specimens were available at birth, 187 (64%) were available for testing at 9 years of age. Of the 202 original enrollees that had both cord and placental tissue specimens available, 156 (77%) were available for testing at 9 years of age. These follow-up rates are comparable with other PCB studies, for example, 71% follow-up for the Collaborative Perinatal Project (Gray et al. 2005) and 68% follow-up for the Lake Michigan study (Jacobson and Jacobson 1996). Neither the participants nor the assessment personnel were aware of the exposure status of the participants.

### Wechsler Intelligence Scale for Children

The Wechsler Intelligence Scale for Children, 3rd ed. (WISC-III; Psychological Corporation, San Antonio, TX; Wechsler 1991) represents the 1991 revision of the most widely used and researched individual measure of children’s intelligence and was the latest available version of the WISC at the inception of this study. It is based on David Wechsler’s view of intelligence as a general or global construct that can be inferred from both verbal and nonverbal performance measures (Matarazzo 1972; Sattler 1992). The WISC-III yields Verbal, Performance, and Full Scale IQ scores along with Verbal Comprehension, Perceptual Organization, Freedom from Distractibility, and Processing Speed Index scores. We calculated Verbal IQ by combining the Information, Similarities, Arithmetic, Vocabulary, and Comprehension subtests. We calculated Performance IQ by combining the Picture Completion, Coding, Picture Arrangement, Block Design and Object Assembly subtests. Index scores are designed to give estimates of factors thought to underlie the WISC-III, and thus aid in the interpretation of children’s performance on the test. To replicate the work of Jacobson and Jacobson (1996) (based on the earlier WISC-R), we administered the Mazes subtest rather than the newly added Symbol Search subtest. As a consequence, we did not compute Processing Speed. All but one subject completed the Freedom from Distractibility testing that subject refused to complete a necessary subtest). We based quantitative interpretation of all scores on a mean (± SD) of 100 ± 15. Internal consistency reliabilities for the Full Scale, Verbal, and Performance IQ scales are excellent (0.91–0.96) and for the Index factor scores range from 0.85 to 0.94. Test–retest reliabilities range from 0.87 to 0.94 for the Verbal, Performance, and Full Scale IQ scores and from 0.82 to 0.84 for Index scores. Construct validity centering on factor analytic methods supports the Verbal and Performance scales of the test (Sattler 1992). The four-factor solution suggested in the manual (Wechsler 1991), underlying the creation of the four Index scores, has received less support in the literature (Bolen 1998). Predictive validity for the WISC-III (which includes data from the WISC-R) reveals strong correlations between the WISC-III and earlier Wechsler tests. IQ–achievement correlations in normal and disabled populations of children appear adequate. The WISC-III norms include females and non-Caucasian groups.

Author E.L., a licensed school psychologist who holds a doctorate in developmental psychology, trained all four WISC-III administrators. We evaluated the following subtests for interrater reliability: Information, Similarities, Arithmetic, Vocabulary, Comprehension, Digit Span, Picture Completion, Coding, Picture Arrangement, Block Design, and Object Assembly. Subtest score interrater reliabilities between one of us (E.L.) and test administrators on four dual-scored WISC-III protocols for each administrator ranged from 0.90 to 1.00. A recent analysis of the effects of rater discrepancies in WISC-III scoring shows those effects to be minimal for broad-cluster and IQ scores (Van Noord and Prevatt 2002). We performed all WISC assessments in Oswego Children’s Study Laboratory our laboratory at the State University of New York at Oswego.

### Classification of exposure

Immediately after birth, we obtained placental tissue (> 20 g) for analysis of organochlorines [PCBs, dichlorodiphenyldichloroethylene (DDE), hexachlorobenzene (HCB), and mirex] by capillary-column gas chromatography (GC) with electron capture detector (ECD). Sample collection and analytic methods have been described previously for all other exposure metrics, including cord blood organochlorines (Stewart et al. 1999, 2000a), hair and placental methylmercury (MeHg) (Magos and Clarkson 1972), cord blood lead (Parsons and Slavin 1993), and postnatal venous lead levels (Gump et al. 2005). Although we collected placental tissues at birth, we kept these samples in an ultracold freezer (−80°C) for nearly a decade. In this article, we report the analysis for placental PCBs, which is the first instance in which this tissue matrix has been available in the Oswego project.

### Analytic methods

PCB analytic methods for placental tissue were peer-reviewed along with this entire report and are included in the Supplemental Material (online at http://www.ehponline.org/members/2008/11058/suppl.pdf). Using GC with ECD and dual-column confirmation, we fully confirmed 75 peaks across both analytic columns [see Supplemental Material (online at http://www.ehponline.org/members/2008/11058/suppl.pdf)]. All congener values were reported by the laboratory and were not censored below detection limits (nor imputed as half the minimum detection limit), which is consistent with our previous work (Stewart et al. 1999, 2000a) and that of others (Fitzgerald et al. 2004; Gray et al. 2005). We quantified only those peaks in which we confirmed the congener on both columns.

### Statistical methods

#### Measurement of potential confounders

We collected data for potential confounding variables from neuropsychological testing instruments, standardized psychometric test batteries, hospital records, structured interviews, and repeated assessments of the home environment [Home Observation for Measurement of the Environment (HOME 1985)] and socioeconomic status (SES). We used several measures of both maternal intelligence and neuropsychological performance in this study. We assessed maternal IQ twice, using the Peabody Picture Vocabulary Test (PPVT) (Dunn 1965) and the Kaufman Brief Intelligence Test (K-BIT) (Kaufman 1990). The correlation between these two IQ measures was *r* = 0.71 (*p* < 0.001). Historically, we have found the average of these two measures to be a stronger predictor of children’s cognitive performance than either alone; we used the average of the two IQ measures as the maternal IQ metric. We also assessed Maternal Color–Word Interference (a measure of cognitive interference control) using the Neurobehavioral Evaluation System 2 (NES2) (Golden et al. 2003). We measured maternal sustained attention and impulsive responding through the use of a Continuous Performance Test (CPT) program (Stewart et al. 2005). We updated Hollingshead SES data with the Hollingshead four-factor socioeconomic scale (Hollingshead 1975). Details regarding the collection of demographic and all other covariate data are given elsewhere (Lonky et al. 1996; Stewart et al. 2000b, 2006). We coded the vast majority of covariates in a continuous fashion and log transformed them (log *x* + 1) if necessary. Variables that were dichotomous included child sex (male/female), child care (preschool, yes/no), and marital status (married, yes/no). These we dummy coded as 0 or 1 in the analysis. Table 2 lists all the covariates considered in the present analysis and their bivariate correlations with outcome measures.

#### Statistical treatment of potential confounders

We controlled for potential confounders using the approach traditionally employed by this investigative team, and then reanalyzed data using an alternative approach as recommended by some reviewers of this report. Method 1 was purely statistical and consistent with that employed previously (Darvill et al. 2000; Stewart et al. 2000b, 2003a, 2003b, 2005), as well as others (Jacobson and Jacobson 1996). Any potential confounding variables even marginally related (*p* < 0.20) to IQ served as covariates in all analyses. We tested each covariate failing to meet the *p* < 0.20 entry criterion (above) to see whether it affected the final outcome of the analysis. Monte Carlo simulations have empirically demonstrated that this additional change-in-estimate criterion, whereby a covariate is added to the equation if it changes the association (β-coefficient) between exposure and outcome by 10% or more, is an effective means of controlling residual bias in multivariate correlational data sets (Maldano and Greenland 1993; Mickey and Greenland 1989). This approach resulted in the statistical control for the following variables: maternal IQ; maternal Wisconsin Card Sort (Heaton 1993), CPT and NES2 performance; parental education; SES; birth order; parental age, weight, and height; the HOME environment; maternal stress and illnesses; birth weight, head circumference, and Ballard score (physical) (Ballard et al. 1991); maternal cigarette smoking and second-hand smoke exposure; DDE levels; maternal tea and caffeine use; child daycare; maternal depression; and marital status. We argue that this approach is conservative because it allows a large number of covariates to account for the data before PCBs are entered, and as a purely statistical approach it is relatively free from investigator bias (Stewart et al. 2005).

Method 2 (a reanalysis) arose from some reviewer critiques suggesting that such a large number of covariates may lead to inefficient models, overcontrol, and possible bias (positive or negative) in the results (Greenland and Robins 1999). To address this, we employed a strategy partly informed by Greenland and Robins (1999) with an emphasis on efficient modeling and a minimal covariate set. We considered only those variables that, based upon the literature, could theoretically function as confounders. This approach eliminated variables that could be anywhere in the exposure–effect causal pathway, such as “parent” variables that could function as a cause of exposure (e.g., years living near the Great Lakes) and “child” variables that could be affected by exposure (e.g., birth weight and head circumference). To further increase model efficiency and eliminate variables that were extraneous (i.e., nonconfounders, not correlated with both exposure and outcome), we included only covariates that were correlated with both exposure and outcome at *p* < 0.20. This approach resulted in the statistical control for the following variables: SES, Maternal Wisconsin Card Sort and NES2 performance, the HOME environment, smoking, and DDE levels. We report the results of method 2 as a check against our traditional approach (method 1) in the analysis with total PCB and IQ.

#### Statistical treatment of the predictor variable (PCBs)

Table 3 shows placental levels of PCBs, DDE, HCB, mirex, and MeHg. One subject had placental PCB levels > 20 ppb. This value was > 10 SDs above the mean and increased the range more than 3-fold. Following the recommendations of Winer (1971), we recoded this outlier to be one point higher than the next highest observed value. Following this, placental PCB levels were normally distributed (skewness = 0.75, kurtosis = 0.47) and not transformed. All other contaminants were similarly normally distributed, and we did not transform them. We assessed the associations between exposure and outcome using a sequential linear regression model. All covariates meeting the criteria above (method 1 in the primary analysis or method 2 for the reanalysis) for entry we entered in the first step, followed by the specific contaminant in the second step. We followed significant findings with regression by dose–response analyses by creating groups at fixed dose intervals, so that the shape of the relationship could be observed graphically. We used two-tailed significance tests (α = 0.05) for inferring a relationship between PCB exposure and IQ.

## Results

Table 4 gives the parameters for the IQ data we obtained, and Table 5 shows the covariate-controlled relationships between PCB exposure and IQ. Results are expressed in standardized regression coefficients (β). Using analysis method 1, total placental PCB was a significant predictor of lower scores on Full Scale IQ (β =−0.167, *p* = 0.021), Verbal IQ (β =−0.213, *p* = 0.003), Verbal Comprehension Index (β =−0.176, *p* = 0.022), and the Freedom from Distractibility scale (β =−0.235, *p* = 0.004). Assuming a linear relationship, in terms of change in whole IQ points per whole unit (ng/g wet weight) of PCBs, expressed using nonstandardized regression coefficients, these results correspond to an estimated drop of 2.9 IQ points, 4.1 Verbal IQ points, 3.3 Verbal Comprehension Index points, and 4.4 Freedom from Distractibility points, all per 1 ng/g PCB. Neither Performance IQ (β =−0.027, *p* = 0.755) nor the Perceptual Organization Index (β =−0.037, *p* = 0.688) was related to exposure. Removal of four children with extremely low IQ (< 70) and two children with extremely high IQ (> 130) did not change the significance of any of the reported findings (Full Scale IQ: β = −0.208, *p* < 0.01; Verbal IQ: β = −0.252, *p* = 0.001; Verbal Comprehension Index: β = −0.215, *p* = 0.009; Freedom from Distractibility: β = −0.258, *p* = 0.004). These results remained significant upon reanalysis using method 2 (Full Scale IQ: β = −0.143, *p* = 0.042; Verbal IQ: β = −0.247, *p* < 0.001; Verbal Comprehension Index: β = −0.191, *p* = 0.011; Freedom from Distractibility: β = −0.210, *p* = 0.003). The specificity of these relationships to placental PCBs was underscored by the fact that all these results remained significant (all *p* < 0.05) even when we added cord blood PCBs, DDE, HCB, and MeHg as covariates. Although total PCBs were significant predictors of IQ overall, analysis of the data using each of the four major PCB congeners (PCBs 118, 138, 153, and 180) indicated that the individual associations with IQ were larger among the more highly chlorinated PCBs (Table 5). In contrast to placental PCBs, cord bloods PCBs were unrelated to IQ. The inability of cord blood alone to predict IQ and/or general cognitive performance has been reported in several other studies (Jacobson and Jacobson 1996; Patandin et al. 1999; Walkowiak et al. 2001).

**Figure 1:**
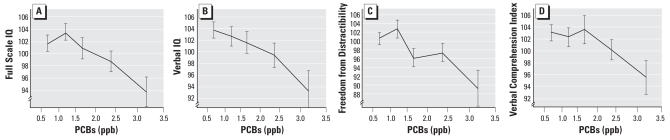
Dose–response functions for PCB–IQ effect expressed in true exposure intervals: Full Scale IQ (*A*), Verbal IQ (*B*), Freedom from Distractibility (*C*), and Verbal Comprehension Index (*D*). Adjusted means ± SE are plotted against the median PCB concentration within each interval, nondetectable to 0.99, 1.00–1.49, 1.50–1.99, 2.00–2.49, and ≥ 2.50 ppb. Linear *F*-tests (Braver and Sheets 1993) showed significant linear dose–response relationships between PCB concentrations and Full Scale IQ, Verbal IQ, and Freedom from Distractibility (all *p* < 0.05).

**Figure 2:**
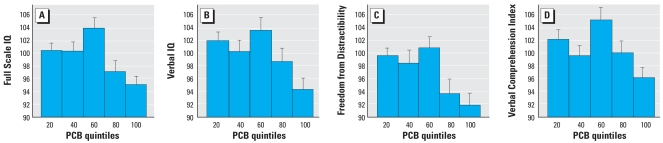
Dose–response functions for PCB–IQ effect expressed ordinally, in exposure quintiles (31–32 subjects per group). Linear *F*-tests (Braver and Sheets 1993) showed significant linear dose–response relationships between PCB concentrations and Full Scale IQ (*A*), Verbal IQ (*B*), and Freedom from Distractibility (*C*) (all *p* < 0.05). (*D*) Verbal Comprehension Index.

We analyzed the shape of the dose–response functions for placental PCBs using polynomial contrasts (trend analyses) across discrete exposure categories (Figures 1 and 2), as a follow-up to the significant linear regressions above. We analyzed the dose–response functions for the PCB effects in two ways. In the first method, we grouped exposure categories on a true concentration basis, with exposure cutoffs as follows: nondetectable to 0.99, 1.00–1.49, 1.50–1.99, 2.00–2.49, and ≥ 2.50 ppb. (Because only 5 subjects had PCB levels < 0.5 ppb, we combined those subjects with the 0.5–0.99 category, resulting in the first interval, nondetectable to 0.99, spanning almost 1 ppb). The advantage of using true exposure intervals (Figure 1) is that the “spacing” between each group can be plotted accurately using the median of each exposure interval, allowing for the most accurate analysis of the dose–response shape. However, the group *n* values were necessarily unequal (*n* = 38, 40, 35, 29, 14). The second method (Figure 2) divided the exposures into quintiles (20th, 40th, 60th, 80th, and 100th percentiles). The advantage of this approach is that it creates essentially equal *n* values (*n* = 31, 31, 32, 31, 31). However, it truncates the natural variability in the data. Nevertheless, the results for both approaches were nearly identical. Using the true concentration intervals, the results indicated significant linearity between PCB exposure and Full Scale IQ (linear *F* = 4.36, *p* = 0.039), Verbal IQ (linear *F* = 5.38, *p* = 0.022), and the Freedom from Distractibility Scale (linear *F* = 6.75, *p* = 0.011). Results were not significant for the Verbal Comprehension Index (linear *F* = 2.50, *p* = 0.117). No quadratic or cubic trends were significant, and we detected no significant departures from linearity using Sidak reversal tests (Braver and Sheets 1993).

Using the percentile groups approach, the results indicated significant linearity between PCB exposure and Full Scale IQ (linear *F* = 5.43, *p* = 0.022), Verbal IQ (linear *F* = 6.27, *p* = 0.014), and the Freedom from Distractibility Scale (linear *F* = 6.89, *p* = 0.010). Results approached significance for the Verbal Comprehension Index (linear *F* = 3.40, *p* = 0.068). No quadratic or cubic trends were significant, and we detected no significant departures from linearity using Sidak reversal tests (Braver and Sheets 1993).

In contrast to PCBs, none of the other contaminants measured in the present study were negatively associated with IQ, excepting one negative association between MeHg and the Freedom from Distractibility Scale (β =−0.171, *p* = 0.050). There was no evidence of an interaction between PCB and MeHg (PCB × MeHg *F* = 0.92, *p* = 0.453). Because MeHg is both neurotoxic and considered an important confounder with PCB exposure, the relationships between PCBs, MeHg, and IQ with and without control for MeHg are shown in Table 6.

Lipid adjustment of PCB levels is a standard way to translate exposure levels both across tissue compartments and across study populations. We did not perform lipid analysis on all placental samples in this study, because an analysis of *n* = 76 randomly selected samples indicated that lipid levels in placenta were exceedingly low (mean lipid = 0.68%), relatively invariant (SD = 0.15%), and uncorrelated with PCB levels (*r* = 0.14, *p* = 0.22). In cases of lipid levels this low, lipid adjustment can compound measurement error and is generally not recommended. Nevertheless, for the simpler purpose of comparing across populations, one can get a general estimate of the lipid-adjusted PCB levels in this cohort by dividing the exposure levels of all subjects by the average lipid concentration (0.68%) generated by the smaller sample from which we derived lipid data. In this case, the median lipid-adjusted PCB level was 221 ng/g fat, and in the highest exposure category 454 ng/g fat. This suggests that the putative PCB effect is occurring at less than half the level (1,250 ng/g) reported by Jacobson and Jacobson (1996).

## Conclusions

The results of the present study support the hypothesis that exposure to PCBs *in utero* is associated with lower IQ in children in the Great Lakes region. Further, as seen in previous studies from the Oswego cohort, the pattern of the relationship between PCB exposure and cognitive functioning is remarkably similar to the pattern seen in PCB-exposed children in Michigan more than a decade ago (Jacobson and Jacobson 1996). Both studies found that prenatal PCB exposure was associated with lower Full Scale IQ, and both found that Verbal IQ, but not Performance IQ, was primarily predictive of the IQ deficit. Moreover, both studies noted that the Freedom from Distractibility scale scores were significantly poorer in PCB-exposed children. In the present study, regression slopes indicated an approximate three-point drop in IQ for every 1 ng/g of PCB exposure. Within the exposure ranges of this study, this translated roughly into a six- to seven-point decline in Full Scale IQ from the least exposed group (average of 0.75 ng/g) to the most highly exposed group (average of 3.15 ng/g PCB). The association was stronger for Verbal IQ, which showed an estimated four-point drop for every 1 ng/g of PCB exposure. This translated roughly into a nine-point drop in Verbal IQ from the least to the most highly exposed groups.

A closer examination of the Freedom from Distractibility and Verbal Comprehension factors can provide some insight into the functional significance between PCB exposure and the associated IQ deficits. The Freedom from Distractibility Index consists of scores from the Arithmetic and Digit Span subtests. Both are considered measures of short-term acquisition and retrieval (Kaufman 1994), reflecting processes of attention, concentration, sequential processing, and short-term memory (Sattler 1992). There is evidence that scores on the Freedom from Distractibility Index are measuring working memory—that is, the ability to hold information in mind to process it (Barkley 1997; Krane and Tannock 2001; Riccio et al. 1997). Although a number of studies have found that lower scores on the Freedom from Distractibility scale are associated with attention deficit hyperactivity disorder (ADHD) (Mayes et al. 1998; Mealer et al. 1996; Snow and Sapp 2000), its utility as a diagnostic identifier of ADHD remains questionable (Reinecke et al. 1999). The Verbal Comprehension Index reflects subtest measures of verbal fluency, knowledge, and comprehension. A strong indicator of general intellectual ability (Sattler 1992), scores on this index are also considered to reflect long-term memory and verbal concept formation.

How confident can we be in the findings linking higher levels of PCB exposure to lower IQ? Epidemiologic studies are always vulnerable to uncontrolled confounding (Stewart et al. 2004), and we submit that the present study has gone to great lengths to address these issues. First, the results we report here are significant even after taking into account and/or statistically controlling for the influence of 52 potential confounding variables. Such control is considerable, even excessive. We used two different approaches to control confounders. We based method 1 (Stewart 2006) on pure empirical relations among the data and favored a maximal covariate set with broad covariate inclusion rule (any covariate related to outcome at *p* < 0.20); method 2 reflected qualitative considerations (eliminating variables that would be irrelevant based on literature or causality considerations), with emphasis on model efficiency and a minimal covariate set. Prenatal PCB exposure significantly predicted lower IQ using either model.

There remains, however, the issue of potential confounding with other non-PCB contaminants in Lake Ontario. This is critical given the numerous organochlorines present in the Great Lakes (Chernyak et al. 2004), as well as the presence of MeHg. We measured several organochlorines typically correlated with PCBs (DDE, HCB, mirex) in this study, and none predicted lower IQ. MeHg did predict poorer performance on the Freedom from Distractibility Scale but, unlike PCBs, was unrelated to Full Scale IQ and Verbal IQ. Further, MeHg levels in the Oswego cohort were extremely low, about a tenth of that seen in the Faroe Islands study, which demonstrated significant, but small, neurobehavioral associations with MeHg (Grandjean et al. 1997). The associations between PCB exposure and IQ were significant after control for MeHg and all other organochlorines. Thus, in this cohort, the relationships between PCB exposure and IQ cannot be readily explained by other contaminants measured.

The potential influence of sample size and possible sampling bias are also issues considered in the present study. The number of subjects with valid IQ and placental PCB data (*n* = 156) was slightly smaller than the number with valid IQ and cord PCB (*n* = 187) data. The number of placental samples was thus 83% of the slightly larger cord blood samples, the latter of which was not associated with IQ. Could it be that the associations between placental PCBs and IQ are spurious due to sampling error? This is highly unlikely. The cord PCB levels in most subjects for whom placental tissues were available were not different from the subjects for whom no placental tissue was available (mean of 1.03 vs. 0.90 ppb, respectively; *t* = 0.65, *p* = 0.51), and cord blood PCBs remained nonsignificant predictors when we restricted the analysis to the pool of subjects who also had placental PCB data available (*n* = 156). Further, the relationship between placental PCBs and IQ remained significant even when applying cord blood PCBs, DDE, and HCB as covariates. When we conducted the analysis only with subjects with both tissue samples analyzed and controlled one for the other, we still observed the association with placental PCBs. The fact remains that placental PCBs were a superior predictor of lower IQ, and the relationship was not an artifact of sampling bias.

Why, then, do placental PCB levels predict impaired IQ, whereas cord blood PCBs do not? This study is not the first in the PCB or MeHg literature to report that different tissue compartments differ in their relationships with cognitive and behavioral outcomes (Grandjean et al. 1997; Jacobson et al. 1990; Patandin et al. 1999; Walkowiak et al. 2001). We only recently completed the analysis of placental tissue for PCBs in this cohort (present results). We designed this analysis to improve exposure assessment through use of the most recent, state-of-the-art PCB analysis and in a tissue matrix that we (correctly) predicted would have fewer nondetectable PCBs than cord blood. With regard to the present study, the most plausible explanation relates to the reliability of the PCB analysis in placenta versus cord blood. PCB levels in placenta were approximately 3-fold higher than in cord blood (median, 0.54 ppb in cord vs. 1.50 ppb for placenta; *F* = 14.05, *p* < 0.0001), and in most instances, the individual PCB congeners were well above the limits of detection. Not so with cord blood, where PCB levels were much nearer the detection limits (Stewart et al. 1999). To place this observation in context, approximately 40% of the sum of highly chlorinated PCBs was detectable in cord blood (Stewart et al. 1999), whereas 100% was detectable in placenta. This range restriction in cord PCBs may explain why the correlation between cord blood and placental PCBs was modest (*r* = 0.39, *p* < 0.001). However, correlations with breast milk PCBs (*n* = 54) were twice as large with placenta (*r* = +0.42, *p* < 0.001) compared with cord (*r* = +0.21, *p* < 0.05), which in terms of *r* translates into four times the predictive power and much higher cross-compartment agreement. These data suggest that in our cohort, PCB exposure in placental tissue may provide a more reliable exposure record than does cord blood. The relationship reported by Jacobson and Jacobson (1996) between PCB exposure and child IQ was not based on cord blood PCBs per se; rather, they maximized the reliability of exposure assessment by including maternal tissue compartments (maternal blood and breast milk) in their exposure metric, an approach somewhat similar to that reported here.

Given the pattern of findings in this study, the question remains: Can we demonstrate a causal relationship between PCB exposure and IQ? Establishing causality in nonexperimental studies is incredibly difficult. As long as unexplained variability remains in a data set, and as long as it is theoretically possible that an unknown confounder could account for the results, we cannot demonstrate causality (Stewart 2006). Although we cannot argue for a causal model, we assert that these data provide strong evidence of a relationship not readily explainable by other factors. If we consider that the association between PCBs and IQ is secondary to a third variable, we must ask: *a*) Why is it that PCB exposure was a significant predictor of lower IQ, independent of > 50 covariates? *b*) Why is it that potentially confounding Great Lakes contaminants, such as MeHg, DDE, and HCB, fail to account for the results? *c*) Why do these data, which show significant deleterious relationships between PCBs and Full Scale IQ, Verbal IQ, and Freedom from Distractibility, replicate so closely the same pattern seen in PCB-exposed children from Lake Michigan a decade ago? It is our view that these questions are difficult to address with arguments of residual confounding or chance relationships. We submit that the data presented in this report represent a reasonable replication of the inverse relationship between PCBs and IQ seen in Lake Michigan (Jacobson and Jacobson 1996 et al. 1996). This strengthens the evidence for a correlation between prenatal and/or perinatal PCB exposure and IQ deficits in children in the Great Lakes region.

## Figures and Tables

**Table 1:** Sample demographic characteristics.^a^

Characteristic	Value
SES^b^ (mean ± SD)	50.8 ± 14.15
Lower class (%)	41.7
Middle class (%)	54.2
Upper class (%)	4.2
Mother married (%)	61.5
Maternal age [years (mean ± SD)]	35.7 ± 4.96
Maternal IQ^c^ (mean ± SD)	102.4 ± 9.68
Child racial characteristics	
White (%)	98.5
African American (%)	1.0
Latin American (%)	0.5
Child sex (% male)	49.0

a Based on all subjects where we measured IQ data (*n* = 187).

b Hollingshead four-factor index.

c Kaufman Brief Intelligence Test (K-BIT).

**Table 2:** Covariate list: Pearson correlation coefficients between covariates and IQ measures.

Covariate	Full Scale IQ	Verbal IQ	Performance IQ	Freedom from Distractibility
Demographic				
Maternal education (years)	0.359#	0.338#	0.285#	0.265#
Paternal education (years)	0.297#	0.304#	0.220#	0.204#
Parity of child (birth order)	−0.146**	−0.159**	−0.120*	−0.101*
SES score (1 year of age)	0.382#	0.349#	0.306#	0.288#
SES score (9 years of age)	0.336#	0.258#	0.341#	0.316#
Maternal IQ [(PPVT + K-BIT)/2]	0.493#	0.442#	0.421#	0.370#
Maternal Wisconsin card sort	0.279#	0.250#	0.238#	0.244#
Maternal sustained attention (Continuous Performance Test)	0.379#	0.342#	0.273#	0.363#
Maternal color–word interference	−0.288#	−0.231#	−0.288#	−0.243#
Maternal height	0.024	0.006	−0.007	0.068
Paternal height	0.164**	0.155**	0.103*	0.048
Paternal age	0.097*	0.107*	0.043	0.102*
Paternal weight	0.104*	0.103*	0.053	0.021
HOME (1 year of age)	0.152**	0.172**	0.089	0.113*
HOME (4.5 years of age)	0.381#	0.387#	0.241#	0.371#
HOME (7 years of age)	0.422#	0.428#	0.273#	0.393#
Maternal age	0.131*	0.145**	0.036	0.187#
Years at address	0.009	−0.002	0.038	0.114*
Years within 50 miles of Great Lakes	0.013	0.017	−0.001	0.082
Child care (preschool)	0.110*	0.095*	0.095*	0.120*
Child home care	0.074	0.078	0.060	0.092*
Maternal depression, current	−0.300#	−0.298#	−0.251#	0.200#
Maternal depression, historical	−0.256#	−0.253#	−0.173**	−0.128*
Married	−0.116*	−0.116*	−0.091	−0.189#
Health/nutrition				
Prepregnancy weight	−0.029	−0.003	−0.750	−0.015
Weight gain during pregnancy	0.059	0.105*	−0.006	0.064
Stress before pregnancy	−0.078	−0.041	−0.078	−0.119*
Stress since learning of pregnancy	0.121*	0.128*	0.081	0.046
Stress in last half of pregnancy	0.066	0.066	0.015	0.049
Maternal illness history	−0.107*	−0.107*	−0.075	−0.072
Obstetric optimality	0.042	−0.021	0.095*	0.048
Vitamins during pregnancy	0.062	0.081	0.017	0.062
Prescription medicines during pregnancy	−0.023	−0.065	0.051	−0.056
Nonprescription medicines during pregnancy	0.045	0.007	0.088	−0.034
Nutrition scale	−0.025	−0.049	0.035	−0.127*
Infant/birth characteristics				
Child sex	−0.011	0.012	−0.023	0.038
Birth weight (g)	0.219#	0.187#	0.173*	0.219#
Head circumference	0.247#	0.186#	0.219#	0.153**
Ballard: neuromuscular	0.080	0.103*	−0.001	0.043
Ballard: physical	0.097*	0.056	0.108*	0.105*
Gestational age at birth	0.027	0.008	0.049	0.006
Erythrocyte porphyrin (cord)	−0.090	−0.115*	−0.053	−0.104*
Maternal substance use				
Cigarettes/day	−0.168**	−0.185#	−0.125*	−0.157**
Second-hand smoke (hr/day)	−0.239#	−0.204#	−0.190#	−0.181#
Alcohol (drinks/day)	0.050	0.083	0.016	0.070
Herbal tea (drinks/month)	0.091*	0.090	0.080	0.099*
Decaffeinated coffee (drinks/month)	0.025	0.035	0.018	0.101*
Diet soda (drinks/month)	0.021	0.012	0.040	0.029
Decaffeinated soda (drinks/month)	−0.004	−0.003	0.031	0.051
Caffeinated beverages (drinks/month)	−0.096*	−0.141**	−0.051	−0.141**
Other contaminants				
Total mercury, first half pregnancy	−0.027	−0.030	−0.028	0.058
Total mercury, second half pregnancy	0.028	0.050	−0.001	0.126*
Placental MeHg	0.241#	0.172**	0.219#	0.150*
Placental DDE	0.163**	0.201**	0.030	0.179**
Placental HCB	0.108*	0.129*	0.038	0.101
Placental mirex	−0.057	−0.067	−0.087	−0.043
Prenatal (cord) lead level	−0.041	0.024	−0.131*	−0.089
Postnatal (blood) lead level	−0.114	−0.160**	−0.063	−0.107

* *p* < 0.20.

** *p* < 0.05.

# *p* < 0.01 (*n* = 187).

**Table 3:** Placental contaminant levels.

		Percentile				
Contaminant	Measure	5th	25th	50th	75th	95th
Total PCBs^a^	ng/g wet	0.54	1.00	1.50	2.06	3.21
	ng/g fat	78.8	147.7	221.0	303.4	473.3
*p*,*p*-DDE	ng/g wet	0.23	0.36	0.54	0.85	1.67
	ng/g fat	33.7	52.2	79.2	124.5	244.8
HCB	ng/g wet	0.04	0.06	0.08	0.10	0.15
	ng/g fat	5.6	9.0	11.2	14.7	22.0
Mirex	ng/g wet	<0.01	<0.01	0.02	0.03	0.07
	ng/g fat	<0.01	1.0	2.2	4.2	10.1
MeHg^b^	ng/g	0.51	1.60	2.52	3.80	5.83

a Placental PCB SD = 0.73 ng/g. We based wet-weight placental PCB levels on the entire sample (*n* = 156) of children for whom placental tissues and IQ data were available. We used a smaller sample (*n* = 76) of placental tissues to estimate the lipid concentration. Lipid levels in placenta were exceedingly low (mean lipid = 0.68%) and relatively invariant (SD = 0.15%). We conducted lipid-based measurements assuming a uniform lipid content.

b Placental MeHg SD = 1.71 (untransformed), 0.200 (log transformed).

**Table 4:** IQ parameters.

Statistic	Full Scale	Verbal	Performance	Freedom from Distractibility
Median	101.0	100.5	100.0	98.0
Mean	99.8	99.9	99.6	97.4
SD	12.7	14.4	13.0	14.1
Range	62–135	57–140	45–127	52–137

**Table 5:** Covariate-controlled relationships between placental PCB levels at birth and IQ at 9 years of age (standardized β-coefficient).

Contaminant	Full Scale IQ (*n* = 156)	Verbal IQ (*n* = 156)	Performance IQ (*n* = 156)	Freedom from Distractibility (*n* = 155)
PCB-118	−0.055 (*p* = 0.461)	−0.111 (*p* = 0.153)	0.039 (*p* = 0.665)	−0.078 (*p* = 0.375)
PCB-138	−0.036 (*p* = 0.665)	−0.102 (*p* = 0.242)	0.061 (*p* = 0.556)	−0.162 (*p* = 0.102)
PCB-153	−0.154 (*p* = 0.064)	−0.234 (*p* = 0.006)	0.001 (*p* = 0.988)	−0.253 (*p* = 0.008)
PCB-180	−0.145 (*p* = 0.057)	−0.207 (*p* = 0.008)	−0.005 (*p* = 0.951)	−0.238 (*p* = 0.006)
Total PCB^a^	−0.167 (*p* = 0.021)	−0.213 (*p* = 0.003)	−0.035 (*p* = 0.682)	−0.235 (*p* = 0.004)
Multiple *R*	*R* = 00.74	*R* = 00.72	*R* = 00.60	*R* = 00.64
Total PCB^b^	−0.143 (*p* = 0.042)	−0.247 (*p* < 0.001)	0.002 (*p* = 0.973)	−0.210 (*p* = 0.003)
Multiple *R*	*R* = 00.61	*R* = 00.58	*R* = 00.49	*R* = 00.53

a Analysis using a maximal covariate set established previously (Stewart et al. 2000b, 2003a, 2003b, 2005, 2006).

b Alternate analysis using a minimal covariate set as recommended by some reviewers.

**Table 6:** Relationships between PCBs, MeHg, and IQ with and without control of each other (standardized β-coefficients).

	Full Scale IQ	Verbal IQ	Performance IQ	Freedom from Distractibility
PCB	−0.167 (*p* = 0.021)	−0.213 (*p* = 0.003)	−0.035 (*p* = 0.682)	−0.235 (*p* = 0.004)
PCB + MeHg controlled	−0.167 (*p* = 0.021)	−0.214 (*p* = 0.003)	−0.040 (*p* = 0.661)	−0.241 (*p* = 0.003)
MeHg	−0.001 (*p* = 0.98)	−0.078 (*p* = 0.367)	−0.001 (*p* = 0.989)	−0.164 (*p* = 0.073)
MeHg + PCB controlled	−0.031 (*p* = 0.70)	−0.059 (*p* = 0.472)	−0.001 (*p* = 0.983)	−0.170 (*p* = 0.050)

Although 156 subjects had placental PCBs available, 145 had both placental PCBs and MeHg available. We based the combined PCB + MeHg analyses on this latter sample.
